# Targeting enhanced cell death represents a potential therapeutic strategy for VEXAS syndrome

**DOI:** 10.1093/rap/rkae065

**Published:** 2024-05-22

**Authors:** Soichiro Adachi, Yohei Kirino, Kana Higashitani, Lisa Hirahara, Ayaka Maeda, Nobuyuki Horita, Kaoru Takase-Minegishi, Ryusuke Yoshimi, Hideaki Nakajima

**Affiliations:** Department of Stem Cell and Immune Regulation, Yokohama City University Graduate School of Medicine, Yokohama, Japan; Department of Stem Cell and Immune Regulation, Yokohama City University Graduate School of Medicine, Yokohama, Japan; Department of Stem Cell and Immune Regulation, Yokohama City University Graduate School of Medicine, Yokohama, Japan; Department of Stem Cell and Immune Regulation, Yokohama City University Graduate School of Medicine, Yokohama, Japan; Department of Stem Cell and Immune Regulation, Yokohama City University Graduate School of Medicine, Yokohama, Japan; Chemotherapy Center, Yokohama City University Hospital, Yokohama, Japan; Department of Stem Cell and Immune Regulation, Yokohama City University Graduate School of Medicine, Yokohama, Japan; Department of Stem Cell and Immune Regulation, Yokohama City University Graduate School of Medicine, Yokohama, Japan; Clinical Laboratory Department, Yokohama City University Hospital, Yokohama, Japan; Department of Stem Cell and Immune Regulation, Yokohama City University Graduate School of Medicine, Yokohama, Japan

**Keywords:** VEXAS syndrome, autoinflammation, *UBA1*, cell death

## Abstract

**Objectives:**

To unravel the mechanisms underlying cell death in the vacuoles, E1 enzyme, X-linked, autoinflammatory, somatic (VEXAS) syndrome using peripheral blood samples and to assess the clinical value of this knowledge.

**Methods:**

Nine patients undergoing treatment for VEXAS syndrome at Yokohama City University Hospital were included in this study. Monocytes and neutrophils were isolated from peripheral blood and then monocytes were differentiated into polarized macrophages. Viable cell counts, cell death assays and measurements of various indicators such as high mobility group box 1 (HMGB1) concentration, extracellular adenosine triphosphate (ATP) concentration, annexin V level and caspase 1, 3 and 7 activities were performed.

**Results:**

Elevated cell death of monocytes and neutrophils was observed in VEXAS syndrome patients, as indicated by cultured cell counts and cell death assays. Annexin V assays and measurements of caspase 1, 3 and 7 activities suggested increased apoptosis and pyroptosis in these cells. Serum HMGB1 levels were significantly elevated in VEXAS syndrome patients and decreased after prednisolone (PSL) dose escalation. Monocytes and neutrophils from the VEXAS group exhibited heightened extracellular ATP secretion, which was significantly reduced by soluble PSL co-culture.

**Conclusion:**

This study confirms increased cell death of monocytes and neutrophils and damage-associated molecular patterns in VEXAS syndrome, and these findings may be valuable for drug screening, therapeutic strategies and as biomarkers.

## GRAPHICAL ABSTRACT

**Figure:**
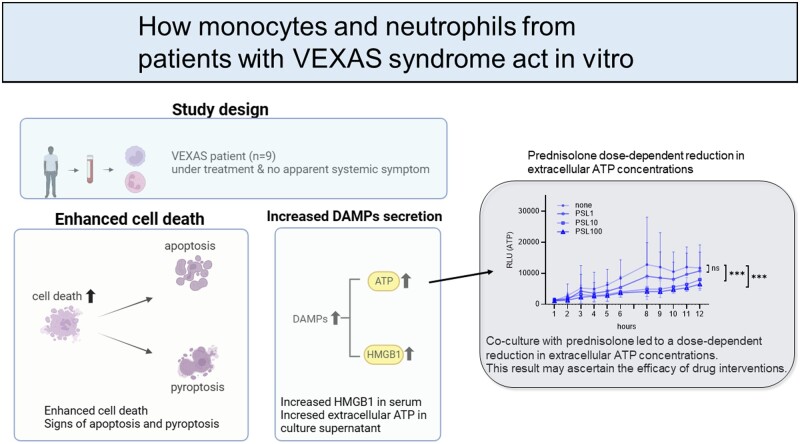

Key messages

- In VEXAS syndrome, elevated cell death in neutrophils and monocytes persists even during clinical remission.
- Prednisolone administration effectively reduced the release of damage-associated molecular patterns from patient monocytes and their serum level.
- Cell death evaluation may be valuable for drug screening, therapeutic strategies and biomarkers for VEXAS syndrome.

## Introduction

VEXAS (vacuoles, E1 enzyme, X-linked, autoinflammatory, somatic) syndrome is a severe autoinflammatory disease caused by acquired somatic *UBA1* variants [1]. *UBA1*, a gene on the X chromosome encoding ubiquitin-like modifier activating enzyme 1, is mutated in myeloid progenitor cells, resulting in decreased UBA1b expression and reduced ubiquitination within the cells [1]. Patients with VEXAS syndrome exhibit inflammation in multiple organs, primarily in the skin, cartilage and lungs [2], and these symptoms often appear after middle age [3–5]. Some cases meet the criteria for recurrent polychondritis (RP) [6], systemic lupus erythematosus (SLE) [7], Behçet’s disease [8], Sweet’s syndrome [9], myelodysplastic syndrome (MDS) [10], multiple myeloma [11], polyarteritis nodosa [12] and giant cell arteritis [13], suggesting heterogeneity of the disease. Biomarkers that can differentiate VEXAS syndrome from various similar inflammatory conditions are required.

Inflammation in VEXAS syndrome is characterized by enhanced innate immunity, marked by the presence of neutrophils in inflamed tissues [14]. Corticosteroids are effective in reducing inflammation in VEXAS syndrome, but patients often require a gradual increase in dosage. The administration of various immunosuppressants, biologics including IL-6 receptor antibodies [15, 16] and Janus kinase inhibitors has been attempted [17], but no alternatives that conclusively spare the prednisolone (PSL) dose are currently available. Allogeneic haematopoietic stem cell transplantation has the potential to cure VEXAS syndrome [18–20], but it is challenging to determine the eligibility for this option given the advanced age of VEXAS syndrome patients, which increases the risk of transplant-related complications. Against this background, alternative drugs to treat VEXAS syndrome are urgently needed.

Single-cell RNA sequencing (sc-RNA-seq) of monocytes from VEXAS syndrome patients has revealed increased signatures of apoptosis, pyroptosis and necroptosis, consistent with PANoptosis, an integrated form of programmed cell death [21]. We therefore hypothesized that measuring cell death and damage-associated molecular patterns (DAMPs) might be clinically useful. While RNA-seq analyses on peripheral blood and bone marrow were conducted in previous studies, *in vitro* experiments using primary monocytes and neutrophils from VEXAS syndrome patients have not been extensively reported. Moreover, detailed investigations into the measurement of DAMPs, crucial in the pathogenesis of various autoimmune diseases [22], have not been documented thoroughly in VEXAS syndrome patients. To address these concerns, we investigated the extent of cell death and DAMPs release in patients with VEXAS syndrome and their clinical significance.

## Methods

### Patients and methods

Patients diagnosed with VEXAS syndrome carrying pathogenic *UBA1* variants were enrolled at Yokohama City University (YCU); some of the clinical data from these patients have been reported previously [6, 23]. The study was approved by the Ethics Committee of YCU Hospital (A121129002) and all participants provided written informed consent before specimen collection. Clinical remission was defined as the absence of symptoms associated with VEXAS syndrome and CRP <5 mg/dl, as modified from a previous study [24]. All healthy controls had no VEXAS-related conditions and no family history. During the course of the study, patients stopped attending the hospital due to death or other reasons, and each experiment was conducted so that at least three patients who were able to continue attending could be recruited at the time the experiment was conducted.

### Isolation of peripheral monocytes and their differentiation into macrophages

Peripheral monocytes and neutrophils were isolated using the EasySep Human Monocyte/Neutrophil Isolation Kit (Stemcell Technologies, Vancouver, BC, Canada). Cell culture was performed in RPMI-1640 (Sigma Aldrich, St Louis, MO, USA) with 10% foetal bovine serum (MP Biomedicals, Santa Ana, CA, USA) and 1% penicillin–streptomycin (Gibco, Waltham, MA, USA). Monocytes (50 000 cells) in 96-well plates were supplemented with 20 ng/ml GM-CSF or 50 ng/ml M-CSF (R&D Systems, Minneapolis, MN, USA) for 7 days, defining GM-CSF-stimulated cells as M1 macrophages (Mφ) and M-CSF-stimulated cells as M2Mφ. The culture medium was changed on days 2, 5 and 7, as previously described [25]. Culture supernatants were collected after 24 hours (monocytes) or 8 hours (neutrophils) and stored at −80°C until use.

### Measurement of cytokines and high mobility group box 1 (HMGB1)

Cytokines in serum were quantified using the LEGENDplex Human Inflammation Panel (BioLegend, San Diego, CA, USA). Serum HMGB1 was measured using the ELISA kit Exp (Sinotest, Kanagawa, Japan) before and after treatment enhancement with PSL.

### Cell counting

M1 or M2Mφ cells on day 7 of culture were air-dried, fixed with 4% paraformaldehyde and stained with the Diff-Quik staining kit (Sysmex Kokusai Shiyaku, Kobe, Japan). The number of Mφ cells per high-power field (200×) was counted using a BX53 microscope (Olympus, Tokyo, Japan).

### Cell death assay

For the cell death assay, 50 000 monocytes and 250 000 neutrophils were seeded in triplicate on a 96-well plate. CellTox Green (CTG) solution (Promega, Madison, WI, USA) was added at 100 μL/well on day 0 and mean fluorescence intensity (MFI) was monitored over time.

### Extracellular adenosine triphosphate (ATP) measurement

For the extracellular ATP measurement, 50 000 monocytes and 250 000 neutrophils were seeded in triplicate on a 96-well plate. RealTime-Glo Extracellular ATP Assay (Promega) was added at 50 μL/well at the start of the experiment and the luminescence intensity was monitored every hour. Referring to a previous report describing that the maximum concentration of PSL in serum was 481 ng/ml (s.d. 81) after a single intravenous injection of 20 mg of sodium PSL succinate in healthy adults [26], we prepared ‘PSL1’ with water-soluble PSL at a final concentration of 480 ng/ml. We then prepared 10-fold and 100-fold concentrations and co-cultured them with monocytes.

### Caspase 3/7, caspase 1 and annexin V assays

Monocytes (50 000/well) and neutrophils (250 000/well) were incubated in 96-well plates for 5 or 24 hours, respectively, and then Caspase-Glo 3/7 or Caspase-Glo 1 (Promega) was added and MFI was measured. The RealTime Glo Annexin V Apoptosis Assay (Promega) was used to obtain data. The time course of annexin V–bound luminescence was evaluated.

### Statistical analysis

GraphPad Prism version 10 (GraphPad Software, La Jolla, CA, USA) and SPSS version 29 (IBM, Armonk, NY, USA) was used for statistical analysis. Student’s unpaired *t*-test and two-way analysis of variance (ANOVA) were performed, with a *P*-value <0.05 being considered statistically significant after adjustment for multiple comparisons.

## Results

### Characteristics of VEXAS syndrome patients included in the study

Nine patients with VEXAS syndrome were recruited in this study. The mean age was 73.1 years (s.d. 11.1). No established diagnostic criteria for VEXAS syndrome are currently available, but all patients had systemic inflammation along with positivity for a pathogenic *UBA1* variant (Table 1). Five patients had MDS, five patients had RP, two patients had large vessel vasculitis and one patient had Sweet’s syndrome. At the time of collection of specimens for *in vitro* experiments, all patients were undergoing treatment with corticosteroids, either alone or in combination with tocilizumab (TCZ) and were considered stable with no apparent systemic symptoms. For patient RP43, blood samples were taken before initiation of PSL or immunosuppressive treatments. Patients and treatments at the time of each experiment are shown in Supplementary Table S1, available at *Rheumatology Advances in Practice* online.

**Table 1.** Demographic characteristics of patients with VEXAS syndrome included in the study

Patient ID	Age of onset, years	*UBA1* variants		VAF, % [23]	MDS [27]	R-IPSS score [28]	VEXAS phenotype	Treatments
RP11	82.8	p.Met41Thr	(c.121A>C)	75.7	MDS-MLD	2.5	RP, pulmonary infiltration	PSL
RP13	66.3	p.Met41Thr	(c.122T>C)	22.4	–	–	RP, skin rash, arthritis	PSL, TCZ
RP15	73.5	p.Met41Thr	(c.122T>C)	68.8	–	–	RP, thrombophlebitis, arthritis	PSL, TCZ
RP16	66.6	p.Met41Lys	(c.121A>C)	87.1	MDS-MLD	3	GCA, RP, DVT, skin rash, arthritis	PSL, TCZ
RP41	64	p.Met41Thr	(c.122T>C)	80.9	MDS-MLD	4.5	RP, Sweet syndrome, pulmonary infiltration, skin rash	PSL, TCZ
RP43	53.8	c.118-1G>C		60.6	MDS-MLD	2–2.5	Skin rash, multiple pulmonary nodules, myocarditis, pleurisy	PSL
RP46	83.8	p.Met41Thr	(c.122T>C)	11.7	MDS-MLD	2–2.5	Sweet syndrome, pulmonary infiltration	PSL, TCZ
RP57	71.2	p.Met41Val	(c.121A>G)	57.3	–	–	Hypertrophic pachymeningitis, IP	PSL
RP99	87	p.Met41Val	(c.121A>G)	62.1	MDS-MLD	0.5	Sweet syndrome, PMR, OP	PSL

Patient IDs are the same as in previous reports [6, 23].

VAF: variant allele frequency; MLD: multilineage dysplasia; R-IPSS: Revised International Prognostic Scoring System; GCA: giant cell arteritis; DVT: deep venous thrombosis; IP: interstitial pneumonia; PMR: polymyalgia rheumatica; OP: organized pneumonia.

### Increased monocyte, neutrophils and monocyte-derived M1/M2 Mφ cell death in VEXAS syndrome

**Figure 1.**
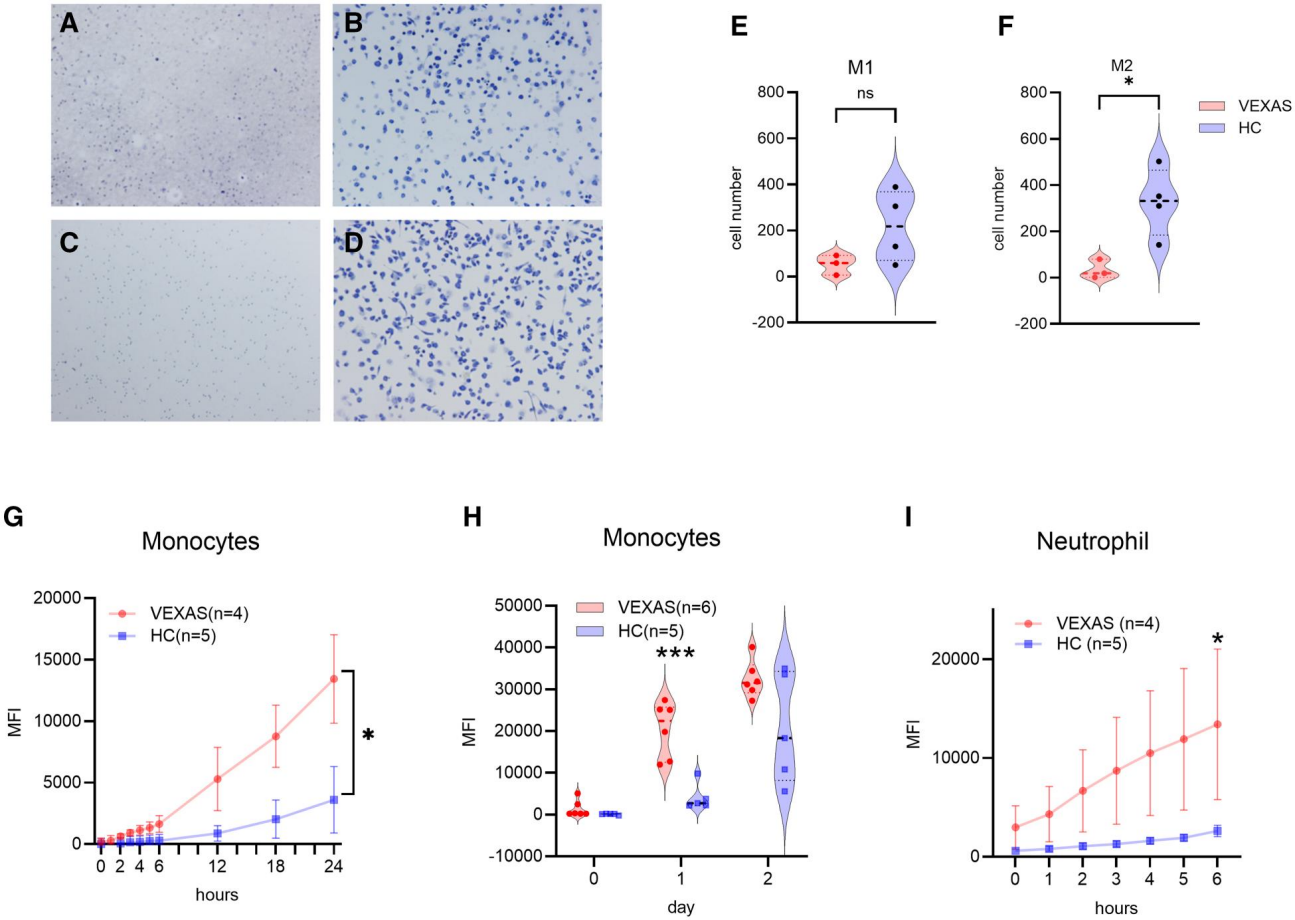
Increased cell death in peripheral monocytes and neutrophils in VEXAS syndrome. Monocytes from four HCs and three patients with VEXAS syndrome stimulated with M-CSF or GM-CSF for 7 days in 96-well dishes to induce M1 and M2 macrophages (Mφ) were stained with Diff-Quik. Representative images of **(A)** M1Mφ and **(B)** M2Mφ of the VEXAS group and **(C)** M1Mφ and **(D)** M2Mφ of the HC group. Comparison of Mφ counts of **(E)** M1 and **(F)** M2 monocytes in HCs and VEXAS syndrome. **(G)** Amount of cell death of monocytes from three HCs and three VEXAS syndrome patients up to 24 h and **(H)** three HCs and six patients from day 0 to day 2 by CellTox Green staining. **(I)** Increased cell death in neutrophils from five HCs and four VEXAS syndrome patients as assessed by CellTox Green staining up to 8 h. *P < 0.05, ***P < 0.001 by unpaired *t*-test or two-way ANOVA

Previous reports have shown that monocyte and neutrophil cell death is increased in VEXAS syndrome [18]. To confirm these findings, we determined the number of Mφs after a 7-day culture period. Consistent with findings in earlier reports, the number of Mφs in the M1 and M2 subsets was decreased in the VEXAS group (Fig. 1A–F). Additionally, cell necrosis was assessed using the CTG assay over time and compared between the VEXAS and healthy control (HC) groups. As depicted in Fig. 1G–I, there were significant increases in the death of both monocytes and neutrophils in VEXAS syndrome. These findings suggest that monocytes and neutrophils in the peripheral blood of patients with VEXAS syndrome exhibit increased susceptibility to cell death.

### Peripheral blood monocytes and neutrophils in VEXAS syndrome are involved in apoptosis and pyroptosis

**Figure 2.**
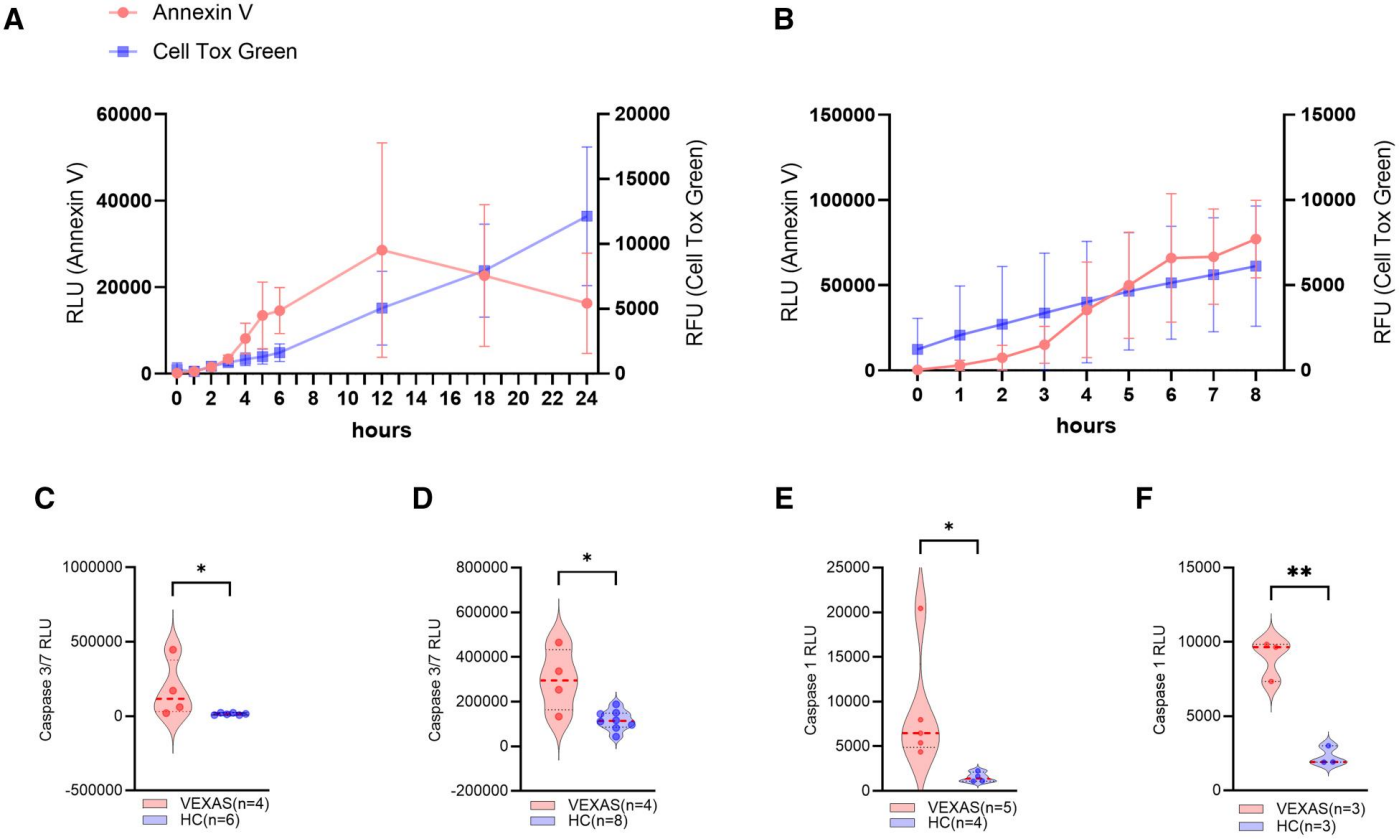
Evaluation of apoptosis and pyroptosis in peripheral monocytes and neutrophils of patients with VEXAS syndrome. **(A, B)** CellTox Green cell death data and RealTime Glo Annexin V apoptosis assay results measured simultaneously are displayed in one figure. **(C, D)** Caspase 3/7 activity and **(E, F)** caspase 1 activity were evaluated in monocytes and neutrophils from HCs and VEXAS syndrome patients. Error bars indicate the s.e.m. *P < 0.05, **P < 0.01 by unpaired *t*-test

To compare the roles of apoptosis and necrosis in VEXAS syndrome, the results of the annexin V assay were integrated with those of the CTG assay: in monocytes, the increase in annexin V (apoptosis) occurred prior to the increase in CTG (necrosis) (Fig. 2A). In contrast, necrosis preceded apoptosis in neutrophils (Fig. 2B). Further investigation of cell death was conducted by analysing caspase 3/7 and caspase 1 activities in the HC and VEXAS groups. Both monocytes and neutrophils showed higher caspase 3/7 activity in the VEXAS group than in the HC group (Fig. 2C and D). Both monocytes and neutrophils displayed predominant caspase 1 activity in the VEXAS group compared with that in the HC group (Fig. 2E and F). These findings suggest the involvement of both apoptosis and pyroptosis in the cell death of peripheral blood leucocytes in individuals with VEXAS syndrome.

### Increased DAMPs in the serum and cells of VEXAS syndrome patients

**Figure 3.**
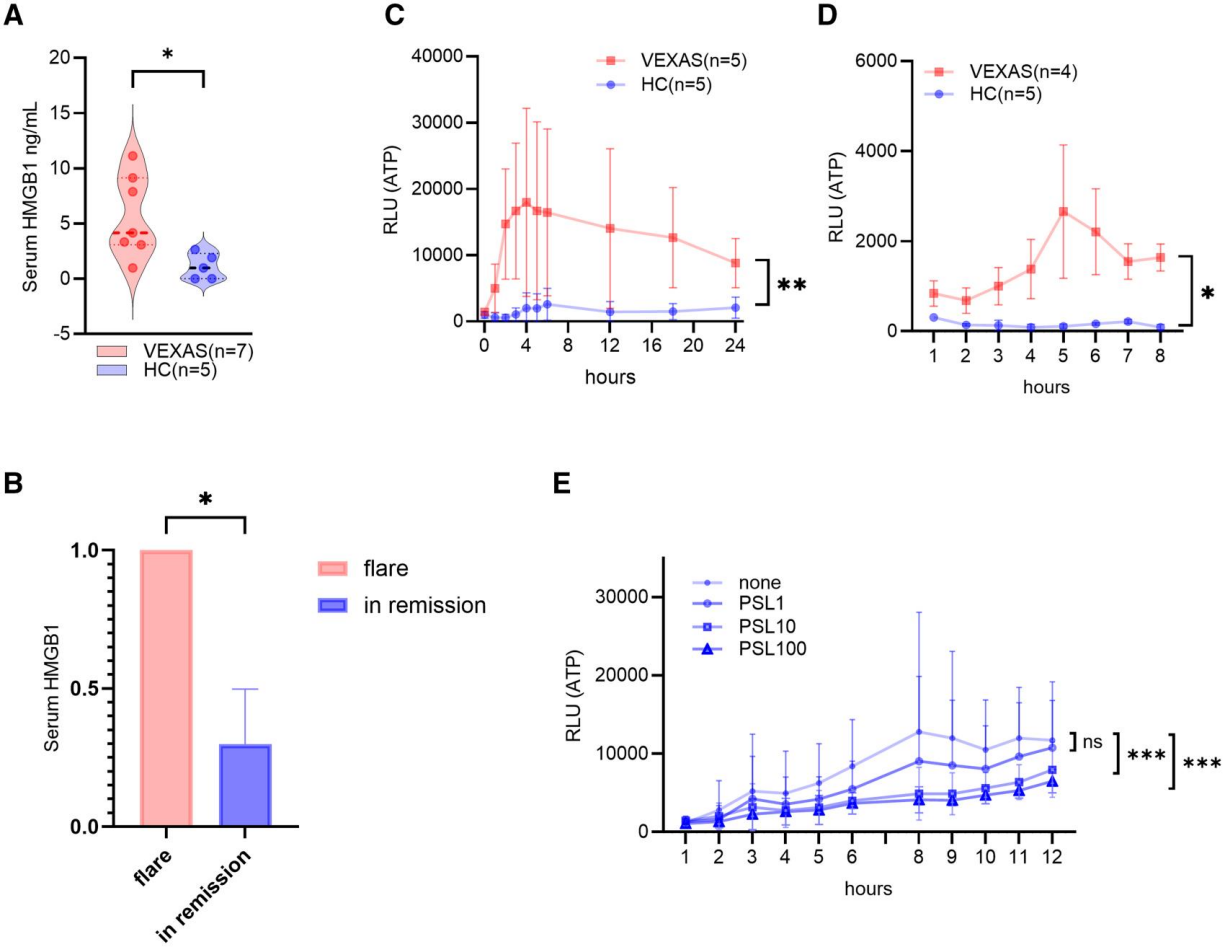
Increased DAMPs in serum and cells of VEXAS patients. **(A)** Serum level of HMGB1 was measured in HCs and patients with VEXAS syndrome in Yokohama City University. **(B)** Relative changes of serum levels of HMGB1 with treatment in relapsed VEXAS syndrome before and after PSL increment. **(C)** Amount of extracellular ATP secretion from monocytes and **(D)** neutrophils from HCs and VEXAS syndrome patients by RealTime-Glo Extracellular ATP Assay. **(E)** Amount of extracellular ATP secretion of monocytes after co-culture with water-soluble prednisolone. Error bars indicate s.e.m. *P < 0.05, **P < 0.01, ***P < 0.001 by unpaired *t*-test or two-way ANOVA or analysis of covariance

The outcomes of the experiments described above confirmed that cell death is elevated in the peripheral blood of patients with VEXAS syndrome. To examine whether DAMPs released due to cell death are indeed elevated in individuals with VEXAS syndrome, the concentration of the cell death marker HMGB1 in patient serum was assessed. The results showed that serum HMGB1 levels in relapsed patients were elevated compared with those in HCs (Fig. 3A). Serum HMGB1 levels decreased following PSL treatment (Fig. 3B and Table 2). Interestingly, serum IL-6 levels decreased rapidly after the increase in PSL dose, while serum IL-18 levels remained high.

**Table 2.** Changes in variables over time in relapsed VEXAS syndrome

Patient ID	Variables	Prior to PSL increment	2 days after PSL increment	4 days after PSL increment
RP41	IL-1β	10.24	0.03	0.32
	IL-6	3056.332	261.466	146.895
	IL-18	3179.77	2149.94	2165.59
	HMGB1	6.191	7.968	0
	CRP	10.2	5.01	0.44
RP99	IL-1β	25.95	0.03	11.44
	IL-6	1713.664	1.598	19.864
	IL-18	2421.15	1316.99	1865.98
	HMGB1	2.1	1.83	0
	CRP	23.21	13.74	3.68
RP43	IL-1β	30.32	1.00	0.09
	IL-6	2200.38	39.49	2.88
	IL-18	5563.73	3968.47	2019.42
	HMGB1	4.609	0.156	0
	CRP	3.41	9.58	1.76
RP16	IL-1β	37.77	7.24	2.62
	IL-6	1057.81	28.21	25.61
	IL-18	11 396.13	6522.73	5971.37
	HMGB1	0	0	0.547
	CRP	38.22	7.48	1.45

Patients in whom cytokine and HMGB1 data were measured simultaneously are listed only if they were measured more than once. Serum IL-1β, 6 and 18 concentrations in units of pg/ml, serum HMGB1 concentration in units of ng/ml, serum CRP concentration in units of mg/dl.

To corroborate the increased release of DAMPs in cultured cells, the extracellular ATP concentration was monitored over time. The results indicated significantly higher extracellular ATP concentrations in the VEXAS syndrome group (Fig. 3C and D). Additionally, co-culture with PSL led to a dose-dependent reduction in extracellular ATP concentrations, bringing them to levels comparable to those in the HC group (Fig. 3E). These findings suggest that the assessment of ATP levels provides a quantitative means of ascertaining the efficacy of drug interventions.

## Discussion

In this study, culture experiments were conducted using monocytes and neutrophils from patients with VEXAS syndrome, with the aim of establishing a foundation for identifying and exploring therapeutic targets. We verified the accelerated cell death of monocytes and neutrophils, innate immune system cells, consistent with previous reports [18]. Furthermore, supplementation with glucocorticoids, which are clinically beneficial in VEXAS syndrome treatment, significantly suppressed the extracellular ATP concentration, suggesting the potential for evaluating the efficacy of new drugs by assessing DAMPs or cell death in *UBA1* mutant cells. This underscores the importance of targeting cell death suppression in future therapeutic strategies in VEXAS syndrome.

Earlier findings demonstrated a high transcriptomic signature of IL-6 in RNA-seq of CD14^+^ monocytes from VEXAS syndrome patients [1]. In addition, sc-RNA-seq of monocytes from patients with VEXAS syndrome revealed increased apoptosis, pyroptosis and necroptosis signatures, consistent with PANoptosis [21], which can induce an IL-6 signature. From a clinical perspective, we reported our experience with the IL-6 receptor agonist TCZ in three patients with VEXAS syndrome. Even in patients who appeared to respond to TCZ, high serum levels of inflammatory cytokines such as IL-1 and IL-18 persisted for >4 months after initiating TCZ [15]. In the current study, leucocytes from patients with no apparent clinical symptoms were used, but increased cell death and secretion of DAMPs persisted. These observations support the hypothesis that PSL and anti-IL-6 receptor antibodies are insufficient to inhibit the cell death that underlies VEXAS syndrome and such cell death may be the basis of the inflammation found in this condition.

In this study, pyroptosis and apoptosis were identified as modes of cell death in VEXAS syndrome monocytes and neutrophils. In haematopoietic cells from VEXAS syndrome patients, mutations in *UBA1* cause abnormal ubiquitination, resulting in the accumulation of proteins that do not fold properly in the endoplasmic reticulum, which is thought to induce the unfolded protein response. The unfolded protein response is known to increase apoptosis [29], and this study confirms that cell death of monocytes and neutrophils is indeed enhanced in peripheral blood. So, determining why *UBA1* mutant haematopoietic cells in peripheral blood rapidly die while those in bone marrow undergo clonal expansion will be important for elucidating therapeutic targets in VEXAS syndrome.

Elevated levels of HMGB1 and extracellular ATP, known as DAMPs [30], were previously observed in VEXAS syndrome patients. HMGB1 is a nuclear protein and, after its translocation into the extracellular lumen during cell activation or cell death, it binds to Toll-like receptors (TLRs) such as TLR2 and TLR4, contributing to the development and onset of autoimmune diseases [31]. Studies have reported that HMGB1 is upregulated in several autoimmune diseases, including rheumatoid arthritis, SLE, type 1 diabetes and autoimmune thyroid disease [32–35]. Serum HMGB1 level may reflect disease activity in VEXAS syndrome, as it decreased with intensified treatment with PSL and should be tested for potential use as a biomarker of this disease.

ATP is released extracellularly following cellular stress and cell death under inflammation, and extracellular ATP signals through membrane-bound purinergic receptors (e.g. P2X4R and P2X7R) [36]. P2X4R and P2X7R are expressed on human macrophages and cause the release of inflammatory cytokines such as IL-1β, IL-6 and TNF-α [37]. P2X7R also results in IL-1β production through NLR family pyrin domain containing 3 inflammasomes [38]. Treatment of cells in the VEXAS group with PSL, which has been clinically proven to be useful, significantly reduced the extracellular secretion of ATP by monocytes, implying that extracellular DAMP concentrations could be used for *in vitro* drug screening.

This study had several limitations, including the small sample size, preventing the comparison of results among different *UBA1* variants, and the heterogeneity in *UBA1* mutant cell frequencies may have affected the extent of cell death. Such variations in *UBA1* mutations in VEXAS syndrome may contribute to distinct phenotypes and disease severity. Indeed, it has been reported that differences in *UBA1* mutations in VEXAS syndrome lead to differences in the translation of UBA1b, which in turn determine the particular phenotype and level of disease severity [39]. Other limitations of this study are the absence of untreated cases and the lack of an analysis of the effects of treatment on cell death *in vivo*.

## Conclusion

This study demonstrates that cell death is enhanced and DAMP levels are increased in patients with VEXAS syndrome and suggests that the *in vitro* evaluation of these features may be helpful for drug screening, therapeutic strategies and biomarkers.

## Supplementary Material

rkae065_Supplementary_Data

## Data Availability

The raw data supporting the conclusions of this article will be made available by the authors, without undue reservation.
