# Supplementary material for: Targeting enhanced cell death represents a potential therapeutic strategy for VEXAS syndrome
Source: Rheumatol Adv Pract. 2024 May 22;8(2):rkae065. doi: 10.1093/rap/rkae065 (PMC11157137; doi:10.1093/rap/rkae065)
Supplement: rkae065_Supplementary_Data [file rkae065_supplementary_data.docx]

Supplementary Table S1. Patient ID numbers used in each chart and the treatment the patients were receiving.

| Figure 1 | RP number | *UBA1* variant | prednisolone | | tocilizumab |
| --- | --- | --- | --- | --- | --- |
| (A-F) | RP13 | p.Met41Thr | 1mg | | 500mg/4 weeks |
|  | RP15 | p.Met41Thr | 12.5mg | | 162mg/10days |
|  | RP16 | p.Met41Lys | 2.5mg | | 480mg/2weeks |
|  |  |  |  | |  |
| (G) | RP13 | p.Met41Thr | 4mg | | 500mg |
|  | RP15 | p.Met41Thr | 10mg | | 162mg/10days |
|  | RP16 | p.Met41Lys | 11mg | | 470/2weeks |
|  | RP46 | p.Met41Thr | 11mg | |  |
|  |  |  |  | |  |
| (H) | RP13 | p.Met41Thr | 4mg | | 500mg/4weeks |
|  | RP15 | p.Met41Thr | 10mg | | 162mg/10days |
|  | RP16 | p.Met41Lys | 10mg | | 470mg/2weeks |
|  | RP43 | c.118-1G>C | none | |  |
|  | RP46 | p.Met41Thr | 11mg | |  |
|  | RP57 | p.Met41Val | 35mg | |  |
|  |  |  |  | |  |
| (I) | RP11 | p.Met41Thr | 15mg | |  |
|  | RP13 | p.Met41Thr | 8mg | | 500mg/4weeks |
|  | RP15 | p.Met41Thr | 8mg | | 162mg/10days |
|  | RP16 | p.Met41Lys | 15mg | | 480mg/2weeks |
|  |  |  |  | |  |
| Figure 2 |  |  |  | |  |
| (A-B) | RP13 | p.Met41Thr | 4mg | | 500mg/4weeks |
|  | RP15 | p.Met41Thr | 9mg | | 162mg/10days |
|  | RP16 | p.Met41Lys | 5mg | | 470mg/2weeks |
|  | RP43 | c.118-1G>C | 15mg | |  |
|  |  |  |  | |  |
| (C) | RP15 | p.Met41Thr | 9mg | | 162mg/10days |
|  | RP16 | p.Met41Lys | 10mg | | 470mg/2weeks |
|  | RP43 | c.118-1G>C | 11mg | |  |
|  | RP99 | p.Met41Val | 15mg | |  |
|  |  |  |  | |  |
| (D) | RP13 | p.Met41Thr | 5mg | | 500mg/4weeks |
|  | RP15 | p.Met41Thr | 8mg | | 162mg/10days |
|  | RP16 | p.Met41Lys | 10mg | | 470mg/2weeks |
|  | RP43 | c.118-1G>C | 11mg | |  |
|  |  |  |  | |  |
| (E) | RP15 | p.Met41Thr | 11mg | | 162mg/2weeks |
|  | RP16 | p.Met41Lys | 10mg | | 470mg/2weeks |
|  | RP41 | p.Met41Thr | 30mg | | 240mg/2weeks |
|  | RP46 | p.Met41Thr | 11mg | |  |
|  | RP99 | p.Met41Val | 14mg | |  |
|  |  |  |  | |  |
| (F) | RP16 | p.Met41Lys | 10mg | | 470mg/2weeks |
|  | RP43 | c.118-1G>C | 20mg | |  |
|  | RP99 | p.Met41Val | 14mg | |  |
|  |  |  |  | |  |
| Figure 3 |  |  |  |  | |
| (A) | RP11 | p.Met41Thr | 10mg |  | |
|  | RP13 | p.Met41Thr | 4mg | 500mg/4weeks | |
|  | RP15 | p.Met41Thr | 10mg | 162mg/10days | |
|  | RP16 | p.Met41Lys | 11mg | 470mg/2weeks | |
|  | RP41 | p.Met41Thr | 25mg | 240mg/2weeks | |
|  | RP43 | c.118-1G>C | none |  | |
|  | RP99 | p.Met41Val | 15mg |  | |
|  |  |  |  |  | |
| (B) | RP13 | p.Met41Thr | 1mg | 500mg/4weeks | |
|  | RP16 | p.Met41Lys | 40mg | 470mg/2weeks | |
|  | RP41 | p.Met41Thr | 20mg | 240mg/2weeks | |
|  | RP43 | c.118-1G>C | 25mg |  | |
|  | RP99 | p.Met41Val | 14mg |  | |
|  |  |  |  |  | |
| (C) | RP13 | p.Met41Thr | 5mg | 500mg/4weeks | |
|  | RP15 | p.Met41Thr | 11mg | 162mg/2weeks | |
|  | RP16 | p.Met41Lys | 7mg | 470mg/2weeks | |
|  | RP46 | p.Met41Thr | 11mg |  | |
|  | RP99 | p.Met41Val | 15mg |  | |
|  |  |  |  |  | |
| (D) | RP13 | p.Met41Thr | 6mg | 500mg/4weeks | |
|  | RP15 | p.Met41Thr | 10mg | 162mg/10days | |
|  | RP16 | p.Met41Lys | 7.5mg | 470mg/2weeks | |
|  | RP46 | p.Met41Thr | 11mg |  | |
|  |  |  |  |  | |
| (E) | RP15 | p.Met41Thr | 11mg | 162mg/2weeks | |
|  | RP16 | p.Met41Lys | 7.5mg | 470mg/2weeks | |
|  | RP43 | c.118-1G>C | 30mg |  | |
|  | RP99 | p.Met41Val | 30mg |  | |
